# 7′-(2,5-Dimeth­oxy­phen­yl)-1′,3′,5′,6′,7′,7a’-hexa­hydro­dispiro­[indan-2,5′-pyrrolo­[1,2-*c*][1,3]thia­zole-6′,2′′-indan]-1,3,1′′-trione

**DOI:** 10.1107/S1600536812003169

**Published:** 2012-02-04

**Authors:** Ang Chee Wei, Mohamed Ashraf Ali, Tan Soo Choon, Ibrahim Abdul Razak, Suhana Arshad

**Affiliations:** a Institute for Research in Molecular Medicine, Universiti Sains Malaysia, Minden 11800, Penang, Malaysia; b School of Physics, Universiti Sains Malaysia, 11800 USM, Penang, Malaysia

## Abstract

In the title compound, C_30_H_25_NO_5_S, all the five-membered rings are in envelope conformations with the spiro and methylene C atoms as the flap atoms. Intra­molecular C—H⋯O inter­actions stabilize the mol­ecular structure and form *S*(6) and *S*(7) ring motifs. The mean plane through the hexa­hydro­pyrrolo­[1,2-*c*]thia­zole ring [r.m.s deviation of 0.0393 (1) Å] makes dihedral angles of 60.92 (5), 88.33 (4) and 84.12 (4)° with the terminal benzene ring and the mean planes of the mono and di-oxo substituted indan rings, respectively. Mol­ecules are linked by inter­molecular C—H⋯O inter­actions into a three-dimensional network. In addition, C—H⋯π and π–π inter­actions [centroid-to-centroid distance = 3.4084 (8) Å] further stabilize the crystal structure.

## Related literature

For related structures, see: Wei, Ali, Choon *et al.* (2011 ▶); Wei, Ali, Ismail *et al.* (2011 ▶); Wei, Ali, Yoon *et al.* (2011 ▶). For ring conformations, see: Cremer & Pople (1975 ▶). For hydrogen-bond motifs, see: Bernstein *et al.* (1995 ▶). For the stability of the temperature controller used for data collection, see: Cosier & Glazer (1986 ▶).
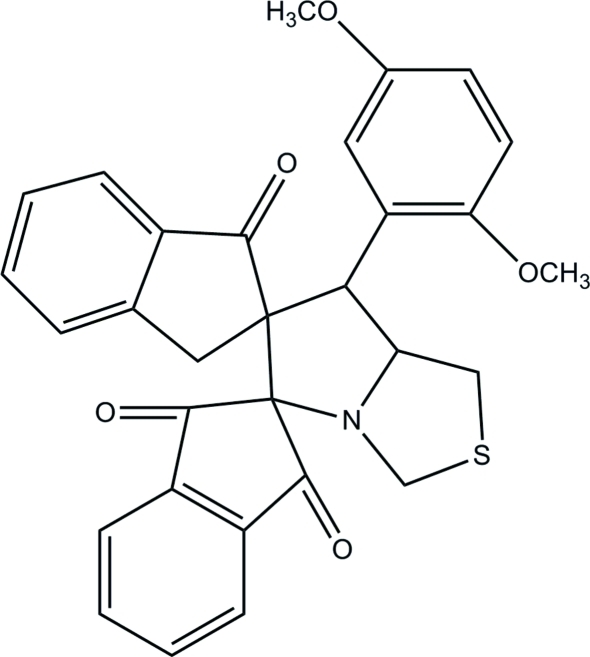


## Experimental

### 

#### Crystal data

C_30_H_25_NO_5_S
*M* *_r_* = 511.57
Triclinic, 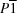

*a* = 9.0425 (4) Å
*b* = 11.1127 (5) Å
*c* = 13.3005 (6) Å
α = 68.016 (1)°
β = 84.588 (1)°
γ = 79.735 (1)°
*V* = 1218.95 (9) Å^3^
*Z* = 2
Mo *K*α radiation
μ = 0.18 mm^−1^
*T* = 100 K
0.36 × 0.19 × 0.10 mm

#### Data collection

Bruker SMART APEXII CCD diffractometer
Absorption correction: multi-scan (*SADABS*; Bruker, 2009 ▶) *T*_min_ = 0.939, *T*_max_ = 0.983
27399 measured reflections
7546 independent reflections
6172 reflections with *I* > 2σ(*I*)
*R*_int_ = 0.030

#### Refinement

*R*[*F* ^2^ > 2σ(*F* ^2^)] = 0.038
*wR*(*F* ^2^) = 0.106
*S* = 1.03
7546 reflections
336 parameters
H-atom parameters constrained
Δρ_max_ = 0.46 e Å^−3^
Δρ_min_ = −0.34 e Å^−3^

### 

Data collection: *APEX2* (Bruker, 2009 ▶); cell refinement: *SAINT* (Bruker, 2009 ▶); data reduction: *SAINT*; program(s) used to solve structure: *SHELXTL* (Sheldrick, 2008 ▶); program(s) used to refine structure: *SHELXTL*; molecular graphics: *SHELXTL*; software used to prepare material for publication: *SHELXTL* and *PLATON* (Spek, 2009 ▶).

## Supplementary Material

Crystal structure: contains datablock(s) global, I. DOI: 10.1107/S1600536812003169/rz2700sup1.cif

Structure factors: contains datablock(s) I. DOI: 10.1107/S1600536812003169/rz2700Isup2.hkl

Additional supplementary materials:  crystallographic information; 3D view; checkCIF report

## Figures and Tables

**Table 1:** Hydrogen-bond geometry (Å, °) *Cg*1 is the centroid of the C16–C21 ring.

*D*—H⋯*A*	*D*—H	H⋯*A*	*D*⋯*A*	*D*—H⋯*A*
C2—H2*A*⋯O1	0.99	2.58	3.2234 (15)	123
C4—H4*A*⋯O1	1.00	2.49	3.1289 (15)	122
C22—H22*B*⋯O2	0.99	2.27	3.0697 (16)	137
C11—H11*A*⋯O3^i^	0.95	2.44	3.1210 (15)	129
C20—H20*A*⋯O2^ii^	0.95	2.48	3.1176 (14)	124
C1—H1*A*⋯O4^iii^	0.99	2.40	3.2806 (16)	148
C30—H30*C*⋯O1^iv^	0.98	2.47	3.2433 (18)	136
C2—H2*B*⋯*Cg*1^v^	0.99	2.58	3.5224 (14)	160

Symmetry codes: (i) 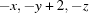; (ii) 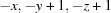; (iii) 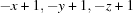; (iv) 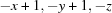; (v) 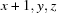.

**Fig. 1.**
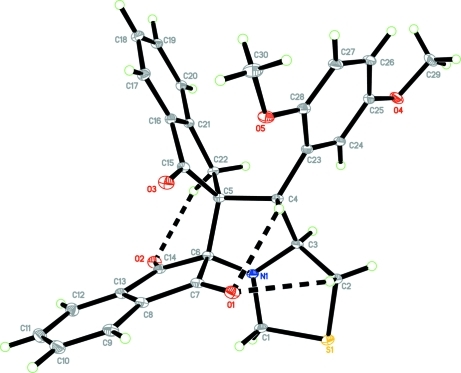
The molecular structure of the title compound, showing 30% probability displacement ellipsoids. Intramolecular hydrogen bonds are shown as dashed lines

**Fig. 2.**
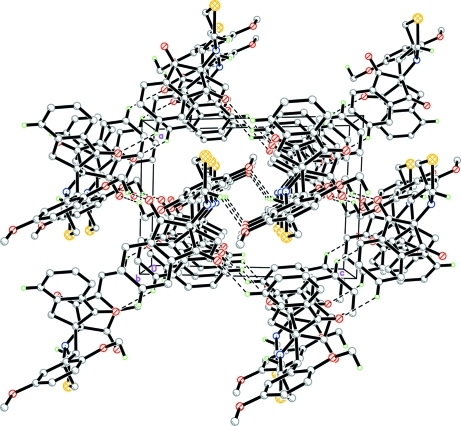
The crystal packing of the title compound viewed along the b axis. The H atoms not involved in intermolecular interactions (dashed lines) have been omitted for clarity.

## supplementary crystallographic information

### Comment

As part of our ongoing search for novel heterocyclic compounds with antitubercular activity (Wei, Ali, Choon *et al.*, 2011; Wei, Ali, Ismail *et al.*, 2011; Wei, Ali, Yoon *et al.*, 2011) our group has synthesized the title compound as described below.

In the molecular structure (Fig. 1), all five-membered rings are in envelope conformation with puckering parameters (Cremer & Pople, 1975) Q = 0.3813 (11) Å and φ = 152.84 (18)° with atom C2 at the flap for the thiazolidine ring (S1/C1/N1/C3/C2), Q = 0.4536 (12) Å and φ = 296.92 (15)° with atom C5 at the flap for the pyrrolidine ring (N1/C3–C6), Q = 0.1467 (12) Å and φ = 357.9 (5)° with atom C5 at the flap for the cyclopentane ring (C5/C15/C16/C21/C22) and Q = 0.0816 (13) Å and φ= 1.5 (9)° with atom C6 at the flap for the cyclopentene ring (C6–C8/C13/C14). The intramolecular C2—H2A···O1, C4—H4A···O1 and C22—H22B···O2 hydrogen bonds (Table 1) stabilize the molecular structure and form *S*(6) and *S*(7) ring motifs (Bernstein *et al.*, 1995). In addition, the mean plane through the hexahydropyrrolo[1,2-*c*]thiazole ring (S1/N1/C1–C6) makes dihedral angles of 60.92 (5), 88.33 (4) and 84.12 (4)° with the terminal benzene ring (C23–C28) and the mean plane of the two 2,3-dihydro-1*H*-indene rings (C5/C15–C22, C6–C14), respectively. The bond lengths and angles are within normal ranges and comparable to related structures (Wei, Ali, Choon *et al.*, 2011; Wei, Ali, Ismail *et al.*, 2011; Wei, Ali, Yoon *et al.*, 2011).

The crystal packing is shown in Fig. 2. The molecules are linked by the intermolecular C11—H11A···O3, C20—H20A···O2, C1—H1A···O4 and C30—H30C···O1 interactions (Table 1) into three-dimensional network. In addition, the crystal structure is further stabilized by an intermolecular C2—H2B···*Cg1* (Table 1) interactions (*Cg*1 is the centroid of the C16–C22 ring). π–π interactions are also observed with centroid to centroid distance *Cg*2···*Cg*3 = 3.4084 (8) Å [*Cg2* and *Cg3* are the centroids of the cyclopentane ring (C5/C15/C16/C21/C22) and cyclopentene ring (C6–C8/C13/C14), respectively].

### Experimental

A mixture of 2-(2,5-dimethoxybenzylidene)-2,3-dihydro-1*H*-indene (0.001 mol), ninhydrin (0.001 mol) and thiazolidine-4-carboxylic acid (0.002 mol) was dissolved in methanol (10 ml) and refluxed for 4 h. After completion of the reaction as evident from TLC, the mixture was poured into crushed ice. The precipitated solid was filtered, washed and recrystallized from petroleum ether–ethyl acetate mixture (1:1 *v*/*v*) to afford the title compound as yellow crystals.

### Refinement

All H atoms were positioned geometrically [C—H = 0.95 and 1.00 Å] and refined using a riding model with *U*_iso_(H) = 1.2 or 1.5*U*_eq_(C). A rotating group model was applied to the methyl groups. Four outliners were omitted from the final refinement, -4 4 6, -1 -3 1, -3 -6 2 and -4 4 7.

### Crystal data

**Table:**

C_30_H_25_NO_5_S	*Z* = 2
*M**_r_* = 511.57	*F*(000) = 536
Triclinic, *P*1	*D*_x_ = 1.394 Mg m^−^^3^
Hall symbol: -P 1	Mo *K*α radiation, λ = 0.71073 Å
*a* = 9.0425 (4) Å	Cell parameters from 9674 reflections
*b* = 11.1127 (5) Å	θ = 2.3–30.7°
*c* = 13.3005 (6) Å	µ = 0.18 mm^−^^1^
α = 68.016 (1)°	*T* = 100 K
β = 84.588 (1)°	Plate, yellow
γ = 79.735 (1)°	0.36 × 0.19 × 0.10 mm
*V* = 1218.95 (9) Å^3^	

### Data collection

**Table:**

Bruker SMART APEXII CCD diffractometer	7546 independent reflections
Radiation source: fine-focus sealed tube	6172 reflections with *I* > 2σ(*I*)
Graphite monochromator	*R*_int_ = 0.030
φ and ω scans	θ_max_ = 30.8°, θ_min_ = 1.7°
Absorption correction: multi-scan (*SADABS*; Bruker, 2009)	*h* = −13→13
*T*_min_ = 0.939, *T*_max_ = 0.983	*k* = −15→16
27399 measured reflections	*l* = −19→19

### Refinement

**Table:**

Refinement on *F*^2^	Primary atom site location: structure-invariant direct methods
Least-squares matrix: full	Secondary atom site location: difference Fourier map
*R*[*F*^2^ > 2σ(*F*^2^)] = 0.038	Hydrogen site location: inferred from neighbouring sites
*wR*(*F*^2^) = 0.106	H-atom parameters constrained
*S* = 1.03	*w* = 1/[σ^2^(*F*_o_^2^) + (0.0547*P*)^2^ + 0.3649*P*] where *P* = (*F*_o_^2^ + 2*F*_c_^2^)/3
7546 reflections	(Δ/σ)_max_ = 0.001
336 parameters	Δρ_max_ = 0.46 e Å^−^^3^
0 restraints	Δρ_min_ = −0.34 e Å^−^^3^

### Special details

**Table:**

Experimental. The crystal was placed in the cold stream of an Oxford Cryosystems Cobra open-flow nitrogen cryostat (Cosier & Glazer, 1986) operating at 100.0 (1) K.
Geometry. All e.s.d.'s (except the e.s.d. in the dihedral angle between two l.s. planes) are estimated using the full covariance matrix. The cell e.s.d.'s are taken into account individually in the estimation of e.s.d.'s in distances, angles and torsion angles; correlations between e.s.d.'s in cell parameters are only used when they are defined by crystal symmetry. An approximate (isotropic) treatment of cell e.s.d.'s is used for estimating e.s.d.'s involving l.s. planes.
Refinement. Refinement of *F*^2^ against ALL reflections. The weighted *R*-factor *wR* and goodness of fit *S* are based on *F*^2^, conventional *R*-factors *R* are based on *F*, with *F* set to zero for negative *F*^2^. The threshold expression of *F*^2^ > σ(*F*^2^) is used only for calculating *R*-factors(gt) *etc*. and is not relevant to the choice of reflections for refinement. *R*-factors based on *F*^2^ are statistically about twice as large as those based on *F*, and *R*-factors based on ALL data will be even larger.

### Fractional atomic coordinates and isotropic or equivalent isotropic displacement parameters (Å^2^)

**Table:**

	*x*	*y*	*z*	*U*_iso_*/*U*_eq_	
S1	0.73773 (3)	0.75887 (3)	0.32247 (3)	0.02242 (8)	
O1	0.48652 (10)	0.81440 (8)	0.08968 (7)	0.02018 (17)	
O2	0.13926 (9)	0.78758 (9)	0.38401 (7)	0.01972 (17)	
O3	0.22209 (10)	0.66773 (8)	0.08718 (6)	0.02009 (17)	
O4	0.69120 (10)	0.08851 (8)	0.50375 (7)	0.02352 (18)	
O5	0.42757 (10)	0.42066 (9)	0.12125 (7)	0.02060 (17)	
N1	0.46129 (10)	0.70280 (9)	0.33292 (8)	0.01505 (17)	
C1	0.53549 (13)	0.81258 (12)	0.32540 (10)	0.0197 (2)	
H1A	0.5058	0.8389	0.3887	0.024*	
H1B	0.5060	0.8890	0.2586	0.024*	
C2	0.71655 (13)	0.62872 (12)	0.27699 (10)	0.0188 (2)	
H2A	0.7143	0.6611	0.1969	0.023*	
H2B	0.8001	0.5546	0.3019	0.023*	
C3	0.56602 (12)	0.58616 (11)	0.32835 (9)	0.01487 (19)	
H3A	0.5821	0.5197	0.4034	0.018*	
C4	0.48564 (12)	0.53056 (11)	0.26182 (8)	0.01420 (19)	
H4A	0.5193	0.5718	0.1844	0.017*	
C5	0.31662 (12)	0.59219 (10)	0.27073 (8)	0.01339 (18)	
C6	0.33703 (12)	0.73236 (11)	0.26064 (8)	0.01379 (19)	
C7	0.36942 (12)	0.82428 (11)	0.14128 (9)	0.0156 (2)	
C8	0.24279 (13)	0.93367 (11)	0.10817 (9)	0.0165 (2)	
C9	0.22010 (14)	1.03660 (12)	0.00841 (10)	0.0206 (2)	
H9A	0.2887	1.0420	−0.0512	0.025*	
C10	0.09331 (15)	1.13115 (12)	−0.00065 (10)	0.0229 (2)	
H10A	0.0747	1.2021	−0.0679	0.028*	
C11	−0.00773 (14)	1.12392 (12)	0.08755 (10)	0.0233 (2)	
H11A	−0.0937	1.1896	0.0789	0.028*	
C12	0.01611 (13)	1.02212 (12)	0.18730 (10)	0.0208 (2)	
H12A	−0.0514	1.0176	0.2474	0.025*	
C13	0.14256 (13)	0.92684 (11)	0.19611 (9)	0.0167 (2)	
C14	0.19522 (12)	0.81249 (11)	0.29325 (9)	0.01488 (19)	
C15	0.22061 (12)	0.59216 (11)	0.18119 (8)	0.01511 (19)	
C16	0.12391 (12)	0.49049 (11)	0.23166 (9)	0.01496 (19)	
C17	0.03024 (13)	0.44286 (12)	0.18221 (9)	0.0189 (2)	
H17A	0.0225	0.4761	0.1056	0.023*	
C18	−0.05095 (13)	0.34581 (12)	0.24801 (10)	0.0209 (2)	
H18A	−0.1146	0.3112	0.2164	0.025*	
C19	−0.03949 (13)	0.29862 (12)	0.36080 (10)	0.0196 (2)	
H19A	−0.0959	0.2322	0.4048	0.024*	
C20	0.05276 (12)	0.34686 (11)	0.40993 (9)	0.0169 (2)	
H20A	0.0591	0.3147	0.4866	0.020*	
C21	0.13569 (12)	0.44365 (11)	0.34392 (9)	0.01400 (19)	
C22	0.24062 (12)	0.51134 (11)	0.37847 (8)	0.01415 (19)	
H22A	0.3162	0.4465	0.4285	0.017*	
H22B	0.1843	0.5694	0.4148	0.017*	
C23	0.52094 (12)	0.38279 (11)	0.29094 (9)	0.01523 (19)	
C24	0.58450 (12)	0.29594 (11)	0.38738 (9)	0.0171 (2)	
H24A	0.5980	0.3275	0.4426	0.020*	
C25	0.62912 (12)	0.16255 (11)	0.40491 (10)	0.0183 (2)	
C26	0.61226 (13)	0.11484 (12)	0.32496 (10)	0.0211 (2)	
H26A	0.6467	0.0252	0.3355	0.025*	
C27	0.54419 (14)	0.19992 (12)	0.22873 (10)	0.0218 (2)	
H27A	0.5302	0.1675	0.1741	0.026*	
C28	0.49688 (13)	0.33129 (11)	0.21227 (9)	0.0175 (2)	
C29	0.75456 (15)	−0.04449 (12)	0.52243 (12)	0.0270 (3)	
H29A	0.7961	−0.0853	0.5951	0.040*	
H29B	0.8348	−0.0474	0.4681	0.040*	
H29C	0.6764	−0.0923	0.5170	0.040*	
C30	0.38015 (16)	0.37160 (14)	0.04664 (10)	0.0272 (3)	
H30A	0.3236	0.4439	−0.0109	0.041*	
H30B	0.3159	0.3050	0.0849	0.041*	
H30C	0.4684	0.3322	0.0147	0.041*	

### Atomic displacement parameters (Å^2^)

**Table:**

	*U*^11^	*U*^22^	*U*^33^	*U*^12^	*U*^13^	*U*^23^
S1	0.01879 (14)	0.01845 (15)	0.03543 (17)	−0.00579 (10)	−0.00349 (11)	−0.01403 (12)
O1	0.0222 (4)	0.0175 (4)	0.0211 (4)	−0.0063 (3)	0.0074 (3)	−0.0077 (3)
O2	0.0224 (4)	0.0204 (4)	0.0179 (4)	−0.0058 (3)	0.0050 (3)	−0.0088 (3)
O3	0.0277 (4)	0.0174 (4)	0.0149 (4)	−0.0050 (3)	−0.0012 (3)	−0.0048 (3)
O4	0.0283 (4)	0.0122 (4)	0.0293 (4)	0.0013 (3)	−0.0056 (3)	−0.0076 (3)
O5	0.0279 (4)	0.0197 (4)	0.0188 (4)	−0.0060 (3)	−0.0003 (3)	−0.0112 (3)
N1	0.0163 (4)	0.0122 (4)	0.0198 (4)	−0.0047 (3)	0.0002 (3)	−0.0085 (3)
C1	0.0189 (5)	0.0160 (5)	0.0294 (6)	−0.0068 (4)	0.0015 (4)	−0.0129 (5)
C2	0.0169 (5)	0.0157 (5)	0.0271 (5)	−0.0047 (4)	−0.0001 (4)	−0.0108 (4)
C3	0.0174 (5)	0.0113 (5)	0.0178 (5)	−0.0040 (4)	−0.0007 (4)	−0.0066 (4)
C4	0.0162 (5)	0.0127 (5)	0.0159 (4)	−0.0053 (4)	0.0019 (3)	−0.0069 (4)
C5	0.0164 (4)	0.0115 (5)	0.0135 (4)	−0.0050 (4)	0.0010 (3)	−0.0051 (4)
C6	0.0155 (4)	0.0121 (5)	0.0148 (4)	−0.0040 (4)	0.0022 (3)	−0.0058 (4)
C7	0.0201 (5)	0.0118 (5)	0.0162 (5)	−0.0057 (4)	0.0028 (4)	−0.0060 (4)
C8	0.0199 (5)	0.0120 (5)	0.0183 (5)	−0.0048 (4)	0.0016 (4)	−0.0058 (4)
C9	0.0255 (6)	0.0163 (5)	0.0196 (5)	−0.0075 (4)	0.0022 (4)	−0.0048 (4)
C10	0.0290 (6)	0.0155 (5)	0.0221 (5)	−0.0043 (4)	−0.0036 (4)	−0.0033 (4)
C11	0.0256 (6)	0.0179 (6)	0.0263 (6)	0.0006 (4)	−0.0039 (5)	−0.0089 (5)
C12	0.0216 (5)	0.0193 (6)	0.0226 (5)	−0.0013 (4)	0.0006 (4)	−0.0099 (5)
C13	0.0190 (5)	0.0135 (5)	0.0188 (5)	−0.0042 (4)	0.0010 (4)	−0.0068 (4)
C14	0.0164 (5)	0.0136 (5)	0.0173 (5)	−0.0054 (4)	0.0019 (4)	−0.0078 (4)
C15	0.0178 (5)	0.0137 (5)	0.0153 (4)	−0.0030 (4)	0.0002 (4)	−0.0068 (4)
C16	0.0155 (5)	0.0143 (5)	0.0168 (5)	−0.0034 (4)	0.0000 (4)	−0.0071 (4)
C17	0.0184 (5)	0.0213 (6)	0.0205 (5)	−0.0034 (4)	−0.0018 (4)	−0.0111 (4)
C18	0.0174 (5)	0.0208 (6)	0.0299 (6)	−0.0051 (4)	−0.0015 (4)	−0.0143 (5)
C19	0.0161 (5)	0.0136 (5)	0.0294 (6)	−0.0049 (4)	0.0005 (4)	−0.0073 (4)
C20	0.0160 (5)	0.0134 (5)	0.0202 (5)	−0.0035 (4)	−0.0001 (4)	−0.0046 (4)
C21	0.0134 (4)	0.0120 (5)	0.0174 (5)	−0.0028 (4)	0.0003 (3)	−0.0061 (4)
C22	0.0170 (5)	0.0136 (5)	0.0133 (4)	−0.0062 (4)	0.0002 (3)	−0.0049 (4)
C23	0.0153 (4)	0.0134 (5)	0.0196 (5)	−0.0051 (4)	0.0031 (4)	−0.0085 (4)
C24	0.0175 (5)	0.0142 (5)	0.0222 (5)	−0.0045 (4)	0.0012 (4)	−0.0092 (4)
C25	0.0166 (5)	0.0138 (5)	0.0256 (5)	−0.0042 (4)	0.0014 (4)	−0.0081 (4)
C26	0.0206 (5)	0.0146 (5)	0.0319 (6)	−0.0050 (4)	0.0030 (4)	−0.0127 (5)
C27	0.0248 (6)	0.0196 (6)	0.0279 (6)	−0.0067 (4)	0.0030 (4)	−0.0159 (5)
C28	0.0187 (5)	0.0172 (5)	0.0201 (5)	−0.0059 (4)	0.0025 (4)	−0.0101 (4)
C29	0.0288 (6)	0.0130 (6)	0.0360 (7)	0.0018 (5)	−0.0007 (5)	−0.0077 (5)
C30	0.0384 (7)	0.0295 (7)	0.0210 (5)	−0.0110 (6)	−0.0008 (5)	−0.0149 (5)

### Geometric parameters (Å, º)

**Table:**

S1—C2	1.8083 (12)	C11—C12	1.3891 (17)
S1—C1	1.8213 (12)	C11—H11A	0.9500
O1—C7	1.2174 (13)	C12—C13	1.3939 (16)
O2—C14	1.2142 (13)	C12—H12A	0.9500
O3—C15	1.2180 (13)	C13—C14	1.4771 (16)
O4—C25	1.3729 (14)	C15—C16	1.4715 (15)
O4—C29	1.4229 (15)	C16—C21	1.3928 (14)
O5—C28	1.3660 (14)	C16—C17	1.3985 (15)
O5—C30	1.4264 (14)	C17—C18	1.3864 (17)
N1—C6	1.4595 (14)	C17—H17A	0.9500
N1—C1	1.4617 (14)	C18—C19	1.3990 (17)
N1—C3	1.4802 (14)	C18—H18A	0.9500
C1—H1A	0.9900	C19—C20	1.3909 (16)
C1—H1B	0.9900	C19—H19A	0.9500
C2—C3	1.5347 (16)	C20—C21	1.3951 (15)
C2—H2A	0.9900	C20—H20A	0.9500
C2—H2B	0.9900	C21—C22	1.5075 (14)
C3—C4	1.5439 (14)	C22—H22A	0.9900
C3—H3A	1.0000	C22—H22B	0.9900
C4—C23	1.5195 (15)	C23—C24	1.3862 (16)
C4—C5	1.5724 (15)	C23—C28	1.4162 (14)
C4—H4A	1.0000	C24—C25	1.4001 (16)
C5—C15	1.5387 (14)	C24—H24A	0.9500
C5—C22	1.5503 (15)	C25—C26	1.3845 (16)
C5—C6	1.5555 (15)	C26—C27	1.3951 (18)
C6—C14	1.5501 (15)	C26—H26A	0.9500
C6—C7	1.5666 (15)	C27—C28	1.3848 (16)
C7—C8	1.4768 (15)	C27—H27A	0.9500
C8—C9	1.3937 (16)	C29—H29A	0.9800
C8—C13	1.3977 (15)	C29—H29B	0.9800
C9—C10	1.3910 (17)	C29—H29C	0.9800
C9—H9A	0.9500	C30—H30A	0.9800
C10—C11	1.4039 (18)	C30—H30B	0.9800
C10—H10A	0.9500	C30—H30C	0.9800
			
C2—S1—C1	92.41 (5)	C8—C13—C14	110.52 (10)
C25—O4—C29	117.86 (10)	O2—C14—C13	125.67 (10)
C28—O5—C30	117.49 (10)	O2—C14—C6	125.75 (10)
C6—N1—C1	116.85 (9)	C13—C14—C6	108.55 (9)
C6—N1—C3	109.94 (8)	O3—C15—C16	127.44 (10)
C1—N1—C3	114.03 (9)	O3—C15—C5	124.96 (10)
N1—C1—S1	107.80 (8)	C16—C15—C5	107.52 (9)
N1—C1—H1A	110.1	C21—C16—C17	121.64 (10)
S1—C1—H1A	110.1	C21—C16—C15	109.33 (9)
N1—C1—H1B	110.1	C17—C16—C15	129.03 (10)
S1—C1—H1B	110.1	C18—C17—C16	118.22 (10)
H1A—C1—H1B	108.5	C18—C17—H17A	120.9
C3—C2—S1	104.63 (7)	C16—C17—H17A	120.9
C3—C2—H2A	110.8	C17—C18—C19	120.29 (10)
S1—C2—H2A	110.8	C17—C18—H18A	119.9
C3—C2—H2B	110.8	C19—C18—H18A	119.9
S1—C2—H2B	110.8	C20—C19—C18	121.43 (11)
H2A—C2—H2B	108.9	C20—C19—H19A	119.3
N1—C3—C2	108.88 (9)	C18—C19—H19A	119.3
N1—C3—C4	105.18 (8)	C19—C20—C21	118.44 (10)
C2—C3—C4	113.74 (9)	C19—C20—H20A	120.8
N1—C3—H3A	109.6	C21—C20—H20A	120.8
C2—C3—H3A	109.6	C16—C21—C20	119.98 (10)
C4—C3—H3A	109.6	C16—C21—C22	112.13 (9)
C23—C4—C3	115.74 (9)	C20—C21—C22	127.88 (10)
C23—C4—C5	117.58 (9)	C21—C22—C5	104.02 (8)
C3—C4—C5	102.55 (8)	C21—C22—H22A	111.0
C23—C4—H4A	106.7	C5—C22—H22A	111.0
C3—C4—H4A	106.7	C21—C22—H22B	111.0
C5—C4—H4A	106.7	C5—C22—H22B	111.0
C15—C5—C22	104.83 (8)	H22A—C22—H22B	109.0
C15—C5—C6	113.28 (9)	C24—C23—C28	117.81 (10)
C22—C5—C6	115.15 (8)	C24—C23—C4	123.85 (9)
C15—C5—C4	112.77 (8)	C28—C23—C4	118.21 (10)
C22—C5—C4	111.13 (8)	C23—C24—C25	121.22 (10)
C6—C5—C4	99.96 (8)	C23—C24—H24A	119.4
N1—C6—C14	112.51 (8)	C25—C24—H24A	119.4
N1—C6—C5	101.36 (8)	O4—C25—C26	124.86 (11)
C14—C6—C5	114.44 (9)	O4—C25—C24	114.81 (10)
N1—C6—C7	113.84 (9)	C26—C25—C24	120.32 (11)
C14—C6—C7	101.90 (8)	C25—C26—C27	119.25 (11)
C5—C6—C7	113.32 (8)	C25—C26—H26A	120.4
O1—C7—C8	125.97 (10)	C27—C26—H26A	120.4
O1—C7—C6	125.21 (10)	C28—C27—C26	120.45 (10)
C8—C7—C6	108.37 (9)	C28—C27—H27A	119.8
C9—C8—C13	121.07 (11)	C26—C27—H27A	119.8
C9—C8—C7	128.86 (10)	O5—C28—C27	124.19 (10)
C13—C8—C7	109.99 (10)	O5—C28—C23	114.99 (10)
C10—C9—C8	117.57 (11)	C27—C28—C23	120.81 (11)
C10—C9—H9A	121.2	O4—C29—H29A	109.5
C8—C9—H9A	121.2	O4—C29—H29B	109.5
C9—C10—C11	121.34 (11)	H29A—C29—H29B	109.5
C9—C10—H10A	119.3	O4—C29—H29C	109.5
C11—C10—H10A	119.3	H29A—C29—H29C	109.5
C12—C11—C10	120.99 (11)	H29B—C29—H29C	109.5
C12—C11—H11A	119.5	O5—C30—H30A	109.5
C10—C11—H11A	119.5	O5—C30—H30B	109.5
C11—C12—C13	117.65 (11)	H30A—C30—H30B	109.5
C11—C12—H12A	121.2	O5—C30—H30C	109.5
C13—C12—H12A	121.2	H30A—C30—H30C	109.5
C12—C13—C8	121.37 (11)	H30B—C30—H30C	109.5
C12—C13—C14	128.06 (10)		
			
C6—N1—C1—S1	−134.10 (8)	C8—C13—C14—O2	173.06 (11)
C3—N1—C1—S1	−3.99 (11)	C12—C13—C14—C6	177.49 (11)
C2—S1—C1—N1	21.48 (9)	C8—C13—C14—C6	−5.04 (12)
C1—S1—C2—C3	−31.84 (8)	N1—C6—C14—O2	−48.10 (15)
C6—N1—C3—C2	113.37 (10)	C5—C6—C14—O2	66.90 (14)
C1—N1—C3—C2	−20.09 (12)	C7—C6—C14—O2	−170.40 (11)
C6—N1—C3—C4	−8.87 (11)	N1—C6—C14—C13	129.99 (9)
C1—N1—C3—C4	−142.33 (9)	C5—C6—C14—C13	−115.01 (10)
S1—C2—C3—N1	34.39 (10)	C7—C6—C14—C13	7.69 (11)
S1—C2—C3—C4	151.30 (8)	C22—C5—C15—O3	162.88 (11)
N1—C3—C4—C23	−149.38 (9)	C6—C5—C15—O3	36.55 (15)
C2—C3—C4—C23	91.57 (11)	C4—C5—C15—O3	−76.08 (14)
N1—C3—C4—C5	−20.04 (10)	C22—C5—C15—C16	−14.21 (11)
C2—C3—C4—C5	−139.09 (9)	C6—C5—C15—C16	−140.54 (9)
C23—C4—C5—C15	−71.71 (12)	C4—C5—C15—C16	106.83 (10)
C3—C4—C5—C15	160.10 (9)	O3—C15—C16—C21	−168.21 (11)
C23—C4—C5—C22	45.66 (12)	C5—C15—C16—C21	8.79 (12)
C3—C4—C5—C22	−82.53 (10)	O3—C15—C16—C17	11.16 (19)
C23—C4—C5—C6	167.69 (9)	C5—C15—C16—C17	−171.85 (11)
C3—C4—C5—C6	39.51 (9)	C21—C16—C17—C18	−0.56 (17)
C1—N1—C6—C14	−71.05 (12)	C15—C16—C17—C18	−179.85 (11)
C3—N1—C6—C14	156.95 (9)	C16—C17—C18—C19	0.60 (17)
C1—N1—C6—C5	166.27 (9)	C17—C18—C19—C20	−0.08 (18)
C3—N1—C6—C5	34.26 (10)	C18—C19—C20—C21	−0.51 (17)
C1—N1—C6—C7	44.24 (12)	C17—C16—C21—C20	−0.02 (16)
C3—N1—C6—C7	−87.76 (11)	C15—C16—C21—C20	179.40 (10)
C15—C5—C6—N1	−164.96 (8)	C17—C16—C21—C22	−178.82 (10)
C22—C5—C6—N1	74.39 (10)	C15—C16—C21—C22	0.60 (12)
C4—C5—C6—N1	−44.74 (9)	C19—C20—C21—C16	0.55 (16)
C15—C5—C6—C14	73.69 (11)	C19—C20—C21—C22	179.14 (10)
C22—C5—C6—C14	−46.96 (12)	C16—C21—C22—C5	−9.50 (12)
C4—C5—C6—C14	−166.09 (8)	C20—C21—C22—C5	171.82 (10)
C15—C5—C6—C7	−42.58 (12)	C15—C5—C22—C21	13.99 (11)
C22—C5—C6—C7	−163.23 (9)	C6—C5—C22—C21	139.16 (9)
C4—C5—C6—C7	77.64 (10)	C4—C5—C22—C21	−108.12 (9)
N1—C6—C7—O1	43.42 (15)	C3—C4—C23—C24	17.73 (15)
C14—C6—C7—O1	164.81 (11)	C5—C4—C23—C24	−103.87 (12)
C5—C6—C7—O1	−71.73 (14)	C3—C4—C23—C28	−157.93 (10)
N1—C6—C7—C8	−129.22 (9)	C5—C4—C23—C28	80.47 (12)
C14—C6—C7—C8	−7.83 (11)	C28—C23—C24—C25	2.52 (16)
C5—C6—C7—C8	115.62 (10)	C4—C23—C24—C25	−173.15 (10)
O1—C7—C8—C9	9.64 (19)	C29—O4—C25—C26	5.59 (17)
C6—C7—C8—C9	−177.79 (11)	C29—O4—C25—C24	−173.03 (11)
O1—C7—C8—C13	−167.21 (11)	C23—C24—C25—O4	179.56 (10)
C6—C7—C8—C13	5.37 (12)	C23—C24—C25—C26	0.87 (17)
C13—C8—C9—C10	−0.76 (17)	O4—C25—C26—C27	178.59 (11)
C7—C8—C9—C10	−177.30 (11)	C24—C25—C26—C27	−2.86 (18)
C8—C9—C10—C11	0.45 (18)	C25—C26—C27—C28	1.39 (18)
C9—C10—C11—C12	0.35 (19)	C30—O5—C28—C27	10.24 (17)
C10—C11—C12—C13	−0.82 (18)	C30—O5—C28—C23	−170.86 (10)
C11—C12—C13—C8	0.51 (17)	C26—C27—C28—O5	−179.08 (11)
C11—C12—C13—C14	177.73 (11)	C26—C27—C28—C23	2.08 (18)
C9—C8—C13—C12	0.29 (17)	C24—C23—C28—O5	177.07 (10)
C7—C8—C13—C12	177.43 (10)	C4—C23—C28—O5	−7.01 (15)
C9—C8—C13—C14	−177.38 (10)	C24—C23—C28—C27	−3.99 (16)
C7—C8—C13—C14	−0.24 (13)	C4—C23—C28—C27	171.93 (10)
C12—C13—C14—O2	−4.42 (19)		

### Hydrogen-bond geometry (Å, º)

Cg1 is the centroid of the C16–C21 ring.

**Table:**

*D*—H···*A*	*D*—H	H···*A*	*D*···*A*	*D*—H···*A*
C2—H2*A*···O1	0.99	2.58	3.2234 (15)	123
C4—H4*A*···O1	1.00	2.49	3.1289 (15)	122
C22—H22*B*···O2	0.99	2.27	3.0697 (16)	137
C11—H11*A*···O3^i^	0.95	2.44	3.1210 (15)	129
C20—H20*A*···O2^ii^	0.95	2.48	3.1176 (14)	124
C1—H1*A*···O4^iii^	0.99	2.40	3.2806 (16)	148
C30—H30*C*···O1^iv^	0.98	2.47	3.2433 (18)	136
C2—H2*B*···*Cg*1^v^	0.99	2.58	3.5224 (14)	160

Symmetry codes: (i) −*x*, −*y*+2, −*z*; (ii) −*x*, −*y*+1, −*z*+1; (iii) −*x*+1, −*y*+1, −*z*+1; (iv) −*x*+1, −*y*+1, −*z*; (v) *x*+1, *y*, *z*.
